# Cancer Evolution Is Associated with Pervasive Positive Selection on Globally Expressed Genes

**DOI:** 10.1371/journal.pgen.1004239

**Published:** 2014-03-06

**Authors:** Sheli L. Ostrow, Ruth Barshir, James DeGregori, Esti Yeger-Lotem, Ruth Hershberg

**Affiliations:** 1 Rachel & Menachem Mendelovitch Evolutionary Processes of Mutation & Natural Selection Research Laboratory, Department of Genetics, the Ruth and Bruce Rappaport Faculty of Medicine, Technion-Israel Institute of Technology, Haifa, Israel; 2 Department of Clinical Biochemistry and Pharmacology, Ben-Gurion University of the Negev, Beer-Sheva, Israel; 3 Department of Biochemistry and Molecular Genetics, Program in Molecular Biology, Integrated Department of Immunology, University of Colorado School of Medicine, Aurora, Colorado, United States of America; University of Washington, United States of America

## Abstract

Cancer is an evolutionary process in which cells acquire new transformative, proliferative and metastatic capabilities. A full understanding of cancer requires learning the dynamics of the cancer evolutionary process. We present here a large-scale analysis of the dynamics of this evolutionary process within tumors, with a focus on breast cancer. We show that the cancer evolutionary process differs greatly from organismal (germline) evolution. Organismal evolution is dominated by purifying selection (that removes mutations that are harmful to fitness). In contrast, in the cancer evolutionary process the dominance of purifying selection is much reduced, allowing for a much easier detection of the signals of positive selection (adaptation). We further show that, as a group, genes that are globally expressed across human tissues show a very strong signal of positive selection within tumors. Indeed, known cancer genes are enriched for global expression patterns. Yet, positive selection is prevalent even on globally expressed genes that have not yet been associated with cancer, suggesting that globally expressed genes are enriched for yet undiscovered cancer related functions. We find that the increased positive selection on globally expressed genes within tumors is not due to their expression in the tissue relevant to the cancer. Rather, such increased adaptation is likely due to globally expressed genes being enriched in important housekeeping and essential functions. Thus, our results suggest that tumor adaptation is most often mediated through somatic changes to those genes that are important for the most basic cellular functions. Together, our analysis reveals the uniqueness of the cancer evolutionary process and the particular importance of globally expressed genes in driving cancer initiation and progression.

## Author Summary

Cancer is a short-term evolutionary process that occurs within our bodies. Here, we demonstrate that the cancer evolutionary process differs greatly from other evolutionary processes. Most evolutionary processes are dominated by purifying selection (that removes harmful mutations). In contrast, in cancer evolution the dominance of purifying selection is much reduced, allowing for an easier detection of the signals of positive selection (that increases the likelihood beneficial mutations will persist). Mutations affected by positive selection within tumors are particularly interesting, as these are the mutations that allow cancer cells to acquire new capabilities important for transformation, tumor maintenance, drug resistance and metastasis. We demonstrate that, within tumors, positive selection strongly affects somatic mutations occurring within genes that are expressed globally, across all human tissues. Fitting with this, we show that genes that are already known to be involved in cancer tend to more often be globally expressed across tissues. However, even when such known cancer genes are removed from consideration, there is significantly more positive selection on the remaining globally expressed genes, suggesting that they are enriched for yet undiscovered cancer related functions. The results we present are important both for understanding cancer as an evolutionary process and to the continuing quest to identify new genes and pathways contributing to cancer.

## Introduction

Cancer initiation and progression are short-term evolutionary processes that occur within our bodies (reviewed in [1]–[5]). A full understanding of cancer requires learning the dynamics of this evolutionary process. All evolutionary processes depend on the existence of genetic variation. In cancer this variation is generated by somatic mutation. The ultimate fate of somatic mutations is affected by natural selection, which acts in two ways: First, it reduces the likelihood that deleterious mutations will persist (purifying selection). Second, it increases the likelihood that functionally advantageous mutations will persist (positive selection). The subset of mutations that persist to the point that we can observe them through DNA sequencing are referred to as substitutions.

Those somatic mutations that are subject to positive selection within tumors are of particular interest, as these are the mutations that contribute positively to transformation, tumor maintenance, expansion, drug resistance, and metastasis. Thus, by inferring what groups of genes are most affected by positive selection within tumors we can gain insight into genes that contribute most positively to the cancer phenotype.

Natural selection affecting somatic mutations acts at the cellular level, in contrast to selection affecting germline (hereditary) mutations which acts at the organismal level. Germline mutations that have a fitness effect are more likely to be deleterious than advantageous because of the complexity of organisms, and because organisms are generally well adapted [6]–[9]. Indeed, it has been shown for many organisms that in germline evolution purifying selection is much more pronounced than positive selection (e.g. [10]–[13]). Much less is understood about how natural selection affects the dynamics of somatic substitution accumulation during cancer initiation and progression.

It is possible to quantify selection by examining patterns of substitution. The ratio of the rates of non-synonymous (change the amino acid sequence) and synonymous (do not change the amino acid sequence) protein-coding substitutions (dN/dS) [14], [15] is the most commonly used metric of selection operating on a system (e.g. [12], [13], [16]–[20]). Since non-synonymous substitutions tend to have a stronger effect on gene function, selection will affect non-synonymous substitutions more often than it affects synonymous substitutions. In the absence of selection, non-synonymous mutations and synonymous mutations will be equally likely to persist (dN/dS ∼1). Purifying selection will more often remove non-synonymous mutations from the population (reducing dN/dS), while positive selection will more often increase their frequency within the population (increasing dN/dS). It is also possible to further classify non-synonymous substitutions as more or less likely to be functional based on considerations of protein sequence conservation (e.g. the SIFT algorithm [21]), or based on protein sequence and structure considerations (e.g. the Polyphen-2 algorithm [22]). Selection is expected to affect more strongly the rates of substitution at more functional (MF) sites than at less functional (LF) sites. Thus, we expect purifying selection to reduce the ratio of the rates of MF and LF substitutions, dMF/dLF, and positive selection to increase dMF/dLF.

Each of these three measures of selection has associated biases and/or limitations. dN/dS may be affected by selection acting on synonymous sites [13], . The SIFT algorithm has a bias by which it is more likely to assign high functionality to residues within proteins that are conserved only over a short evolutionary distance, but are highly similar within this short time-frame [21]. The Polyphen algorithm has a bias by which its likelihood of assigning functionality to a mutation is higher, if the mutated allele happens to be the allele represented in the human reference genome [25]. Each of these biases may affect the results obtained by any one measure. By combining and contrasting results obtained using the three measures we can examine whether patterns we observe are more likely to be truly significant.

Many past studies have used dN/dS to search for genes under strong positive selection in both organismal evolution and within tumors (e.g. [26]–[29]). Such studies have attempted to identify genes for which dN/dS is significantly higher than 1. It is important to note however that this is an extremely conservative test for positive selection [14], [30]. After all, in order for dN/dS to reach values significantly higher than 1, positive selection would have to be strong enough to overcome the contradictory action of purifying selection, which acts to reduce dN/dS. It is quite likely that many genes that are subject to positive selection will have dN/dS values equal or lower than 1 due to the fact that they are also subject to strong purifying selection.

Here we suggest an alternative approach for identifying whether a group of genes is subject to positive selection within tumors. In this approach we identify a group of genes, which we can demonstrate is enriched for important functionality, and therefore subject to stronger purifying selection in the germline. If we can then show that when examining cancer somatic substitutions these genes have higher dN/dS and dMF/dLF values than other genes, such increased values are very unlikely to be explained by more relaxed purifying selection acting on such important genes. Rather, higher cancer somatic functional variation within more important genes is likely to represent increased positive selection. This method allows us to detect positive selection on important genes even if such positive selection does not lead to dN/dS and dMF/dLF values that are higher than 1.

We used extensive data of cancer somatic substitutions to examine the directionality and intensity of selection acting within tumors. We find that natural selection affects somatic mutations within tumors in a much different manner compared to the way it affects germline mutations. More specifically, somatic mutations within tumors are subjected to much more relaxed purifying selection, and to much more pronounced positive selection relative to germline mutations. Positive selection is particularly strong within tumors on genes that are expressed globally across human tissues. Indeed, we show that known cancer genes (which we know to be positively selected in tumors) are highly enriched for global expression patterns, and substantially depleted for tissue-specific expression patterns, compared to non-cancer associated genes. Yet, even if known cancer genes are removed from consideration, we can still detect stronger positive selection on the remaining globally expressed genes, suggesting that globally expressed genes are enriched for yet undiscovered cancer related functions.

## Results

### The proportion of functional substitutions within breast tumors is much higher than in the germline

**Figure 1:**
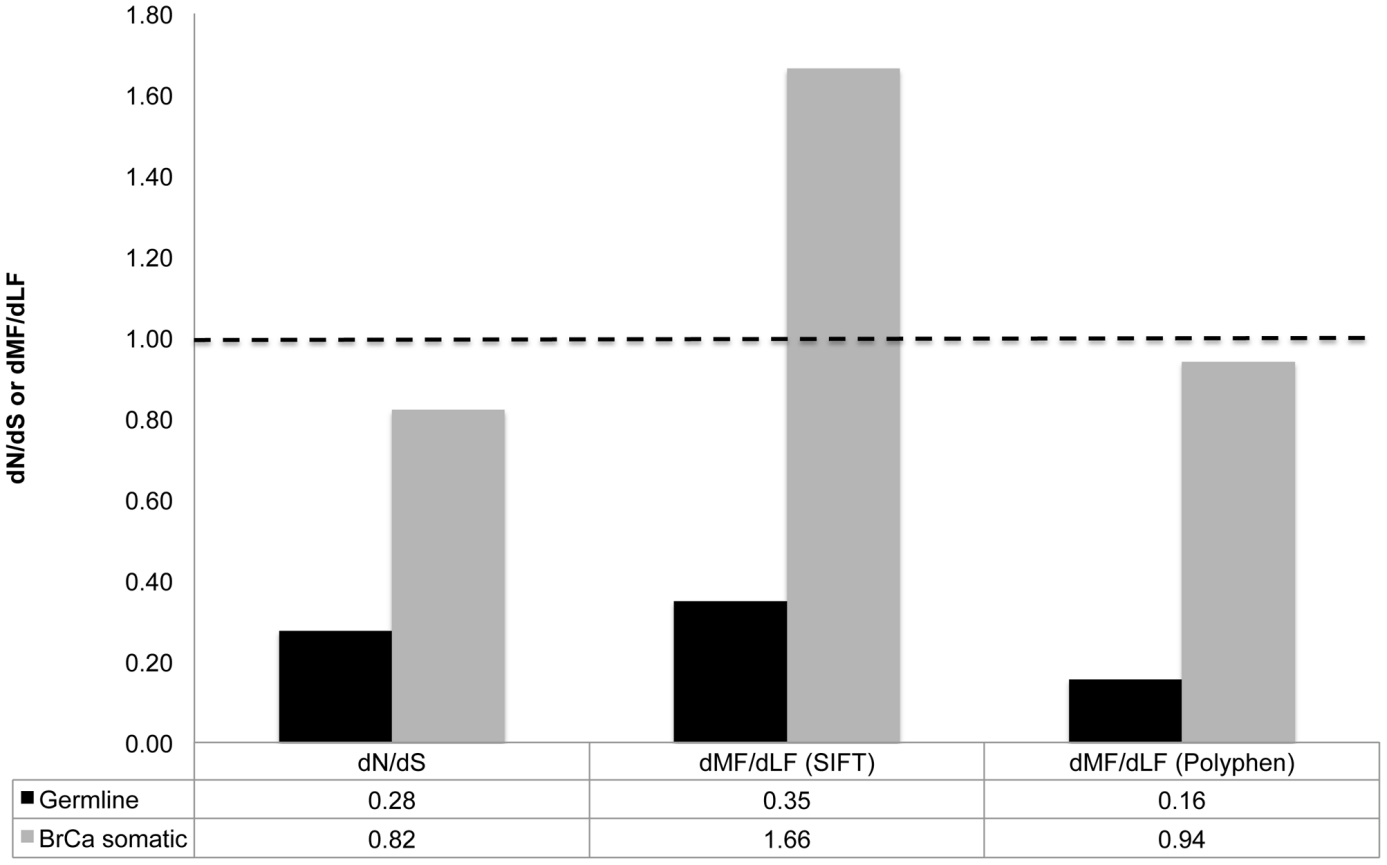
Increased proportion of functional substitutions in BrCa compared to the germline. Depicted are dN/dS and dMF/dLF values calculated based on germline mutations segregating at a frequency of >0.1 (black), and dN/dS and dMF/dLF values calculated based on BrCa somatic substitutions (gray). The dashed line represents a dN/dS and dMF/dLF ratio of 1. We focus on germline substitutions occurring at a higher frequency of >0.1 in the human population, because rare germline substitutions are expected to be less affected by natural selection [51]. This is because rare polymorphisms have not yet had time to be strongly affected by selection and therefore still contain many deleterious substitutions that with time would be removed from the population. The full data regarding numbers of non-synonymous, and synonymous, MF and LF substitutions, and also regarding dN/dS and dMF/dLF of all germline substitutions (including those appearing at frequencies lower than 0.1) is presented in Table S1.

We calculated dN/dS, dMF/dLF(SIFT) and dMF/dLF(Polyphen-2) for germline and breast cancer (BrCa) somatic substitutions. Germline data were extracted from the 1000 human genome project [11], and data of BrCa somatic substitutions were extracted from The Cancer Genome Atlas (TCGA) project [31]. We found that dN/dS and dMF/dLF of BrCa somatic substitutions are much higher than observed for germline substitutions (Figure 1, Table S1). These results are consistent with the results of a previous study that examined dN/dS in four other tumor types and found elevated values [17]. That study attributed these elevated dN/dS values to a sharp relaxation in purifying selection. Indeed, it makes intuitive sense that purifying selection on somatic substitutions should be relaxed, compared to its effect on germline mutations, because somatic mutations affect only the cells in which they occur and their progeny, while germline mutations affect the entire organism. Thus, many deleterious mutations that would be affected by purifying selection in the germline may not be subject to such selection when they occur as somatic mutations in a tissue in which the gene they affect is not active. Additionally, the efficiency of selection on moderately deleterious mutations in tumors may be reduced due to hitchhiking and the effects of Muller's ratchet [32]. Small effective population sizes of stem cell pools may increase the power of genetic drift, relative that of selection, which would further reduce the efficacy of selection. Interestingly, both dN/dS and dMF/dLF(Polyphen) are significantly lower than 1 for BrCa somatic substitutions (*P*<<0.0001), indicating that somatic substitutions in breast tumors remain significantly affected by purifying selection, albeit weakly. At the same time dMF/dLF(SIFT) is significantly higher than 1 (*P*<<0.0001), indicating a strong effect of positive selection on breast cancer somatic substitutions. It is important to note that each of the three measures of selection may have intrinsic biases (see Introduction). We are therefore hesitant to draw any conclusions that are not supported by at least a majority of the measures used.

### Very few known cancer genes show a clear signal of positive selection of dN/dS higher than 1

Since cancer is a process of cellular adaptation in which cells acquire the capability to proliferate more efficiently and gain additional functions, it is reasonable to expect that a significant number of BrCa somatic mutations are under positive selection. Increased dN/dS and dMF/dLF values can stem not only from reductions in purifying selection but also from increases in positive selection. As discussed in the Introduction, the most commonly used methodology to detect positive selection is to examine whether dN/dS values for a certain gene or group of genes are significantly greater than 1. To estimate how well this method would be expected to work in detecting positive selection within tumors, we calculated dN/dS for each gene within the human genome, based on data of somatic cancer substitutions extracted from the TCGA. To maximize our ability to detect positive selection, gene-by-gene, by maximizing the number of substitutions available for each gene, we combined data of all 16 tumor-types available within the TCGA, for which no publication restriction applied as of the end of December 2013 (Table S2). This allowed us to calculate dN/dS for 18,299 human genes, in which at least one synonymous substitution was observed in the entire dataset. Of these genes 456 have been causatively implicated in cancer according to the ‘cancer gene census’ database [33]. Such known cancer genes should be subject to positive selection in cancer evolution, as they carry mutations that allow cells to gain the proliferation and invasion capabilities needed to develop and maintain a tumor, and for its metastasis. Of the 18,299 genes for which we could calculate dN/dS only 104 had values significantly greater than 1 (*P*<0.05 according to a χ2 test, Table S3). Seventeen of these (16.3%) were known cancer genes (a significant enrichment, *P*<<0.0001 according to a χ2 test). This is consistent with previous results that have shown that the known cancer genes, contained within the cancer gene census, are more likely to show a significant signal of positive selection [32]. At the same time, 439 of the 456 cancer genes (96.3%) did not have dN/dS values significantly greater than 1. It is therefore apparent that attempting to identify genes under positive selection within tumors by requiring dN/dS to be higher than 1 would fail to identify the vast majority of genes that are subject to positive selection within tumors.

A similar conclusion can be reached when considering only those cancer genes that have been implicated specifically in breast cancer, and considering only BrCa somatic substitutions. Of the 15 genes contained in the cancer gene census for which somatic mutations were implicated in BrCa, only two (TP53, and PIK3CA) present dN/dS values significantly higher than 1. It therefore becomes apparent that in order to identify many genes that are important for cancer and thus evolving under positive selection within tumors one must devise more sensitive means. This becomes even more apparent when one considers that of the 772 BrCa tumors for which there is available sequence data, 360 (46.6%) have no somatic mutations in the 15 genes already implicated in BrCa. This strongly suggests that there are multiple BrCa drivers yet undiscovered.

It is important to note that our results do not imply that looking for genes with dN/dS significantly greater than 1 is not a useful approach. Indeed this approach has allowed us to identify some good candidates for involvement in cancer that have not been previously implicated in cancer, according to the cancer gene census (Tables S3 and S4). When looking for genes in which dN/dS of BrCa somatic substitutions is significantly higher than 1 (Table S4), we find two genes that are not contained in the cancer gene census database. These are the Titin gene, TTN, and MLL3, which has been associated with other types of cancer, but not with BrCa. These two genes may be good candidates for involvement in BrCa. Additionally, 87 of the 104 genes we identified as having dN/dS significantly greater than 1, based on combined data of somatic cancer substitutions from 16 types of tumors (Table S3) have not been implicated in any type of cancer, according to the cancer gene census. These genes may also provide good novel candidates for involvement in cancer. Combined, these results show that screening for genes for which dN/dS is significantly greater than 1 may indeed identify some novel genes under positive selection within tumors. However, this method lacks sensitivity and misses much of the positive selection occurring within tumors.

### Higher proportion of functional BrCa somatic substitutions within globally compared to non-globally expressed genes

The results presented above demonstrate that a signal of positive selection of dN/dS significantly higher than 1 can only be obtained for a very small minority of cancer genes. This likely stems from the fact that both positive and negative selection affect dN/dS of genes in consort. While positive selection increases dN/dS, purifying selection pushes it down. In order to allow us to detect positive selection acting within tumors with higher sensitivity, we devised a different approach. The idea behind this approach is to identify instances in which higher levels of somatic functional variation are observed within genes that are more important and more constrained at the germline level, compared to less important, less constrained genes. Since more important, more constrained genes are expected to be subject to stronger purifying selection, higher levels of functional variation within such genes will likely reflect the action of positive, rather than purifying selection.

We considered separately two groups of genes: those globally expressed across 16 examined tissues (Materials and Methods), and those whose expression is restricted to only a few or to none of these tissues. We have four lines of evidence that globally expressed genes are more important than non-globally expressed genes, and are likely evolving under increased constraint. First, globally expressed genes are enriched for important housekeeping functions [34]. Second, we found that globally expressed genes are significantly more likely to be predicted as essential, compared to non-globally expressed genes (*P*<<0.0001, according to a to a χ2 test, based on data extracted from [35] (Materials and Methods)). Third, it has been previously demonstrated, using data of sequence divergence between humans and rodents, that rates of non-synonymous substitution are almost threefold lower for globally expressed genes, compared to genes with tissue-specific expression patterns [36]. Fourth, we found that for human polymorphism data, dN/dS of globally expressed genes is significantly lower than that of non-globally expressed genes (0.2 vs. 0.28 for germline substitutions appearing at a frequency of >0.1, *P*<<0.0001), directly demonstrating stronger purifying selection on globally expressed genes. dMF/dLF(Polyphen) is also significantly (albeit only marginally so, *P* = 0.05) lower for globally expressed genes, when data of human polymorphism is considered (0.13 for globally expressed genes vs. 0.15 for non-globally expressed genes). This again indicates that there is stronger constraint acting on germline mutations occurring within globally expressed genes. No significant difference in human polymorphism dMF/dLF(SIFT) is observed between globally and non-globally expressed genes (*P* = 0.16).

In sharp contrast to the increased constraint acting on globally expressed genes in the germline, dN/dS of globally expressed genes in BrCa tumors is significantly higher than that of non-globally expressed genes (Table 1, *P*<<0.0001). This difference remains consistent and even intensifies when the BrCa dN/dS of globally expressed genes is compared to that of non-globally expressed genes that are not expressed in breast (Table 1, *P*<<0.0001). As demonstrated above, globally expressed genes are expected to be subject to more, rather than less, selection than the “average” gene, and BrCa somatic mutations occurring in genes not expressed in breast tissue should be under the weakest selection of all. Therefore, higher somatic dN/dS values in globally expressed genes are unlikely to reflect weaker purifying selection on these genes. Rather, such higher dN/dS values likely reflect stronger positive selection acting on globally expressed genes than on non-globally expressed genes in BrCa. Similarly, we find that dMF/dLF (SIFT) and dMF/dLF(Polyphen-2) are significantly higher for somatic BrCa substitutions in globally expressed genes than in non-globally expressed genes (Table 1, *P*<<0.0001, for all comparisons). We observe this result even with a less stringent threshold for globally expressed genes of expression in 14–16 tissues (Table S5, *P*<<0.0001 for all comparisons). It is important to note that these results indicate that positive selection on globally expressed genes is very strong. After all, we are able to observe its signal even in the face of an opposite force (purifying selection) that almost certainly acts to remove non-synonymous and more functional somatic substitutions more efficiently from globally expressed genes.

**Table 1:** Globally expressed genes are enriched for functional BrCa somatic substitutions compared to genes that are not globally expressed.

	Non-synonymous vs. synonymous			SIFT			Polyphen		
	# non-syn	# syn	dN/dS	# MF	# LF	dMF/dLF	# MF	# LF	dMF/dLF
Globally expressed genes	10653	3463	0.91	5960	4355	1.91	5252	4415	1.01
Non globally expressed genes	18014	6911	0.78	9210	7906	1.55	7922	7779	0.91
Non-globally expressed genes, expressed in breast	8863	3275	0.81	4706	3937	1.60	4149	3866	0.94
Non-globally expressed genes, not expressed in breast	9151	3636	0.76	4504	3969	1.51	3773	3913	0.88
Globally expressed genes, known cancer genes removed	9623	3310	0.86	5202	4091	1.77	4616	4074	0.97
Non globally expressed genes, known cancer genes removed	17580	6765	0.78	8972	7717	1.55	7719	7592	0.91
Cancer associated genes^a^	1504	318	1.39	1026	463	3.08	851	537	1.34
Genes not yet associated with cancer^a^	29022	10804	0.80	14828	12423	1.61	12718	12125	0.92

a According to the Catalogue of Somatic Mutations in Cancer (COSMIC) [33]

Interestingly, dN/dS and dMF/dLF of globally expressed genes are also significantly higher when compared only to non-globally expressed genes that are expressed in breast tissue (Table 1, *P*<<0.0001 for dN/dS and dMF/dLF(SIFT), *P* = 0.01 for dMF/dLF(Polyphen-2)). Thus, the increased positive selection on globally expressed genes in breast tumors cannot be due solely to their expression in breast.

### Globally expressed genes are enriched for functional somatic substitutions in many additional cancer types

We examined whether our findings of increased functional variation of cancer somatic compared to germline substitutions, and of increased functional variation in globally expressed compared to non-globally expressed genes extend to additional tumor types. We analyzed data of somatic substitutions in 13 additional types of tumors, for which at least 5000 somatic non-synonymous and synonymous substitutions were available in the TCGA dataset, and for which there were no publication restrictions as of the end of December 2013 (Table 2 and Table S2). Similarly to what we found for BrCa, dN/dS and dMF/dLF values tend to be much higher for somatic compared to germline substitutions across all tumor types (Table 2). This indicates that purifying selection is relaxed on all types of tumors. Also consistent with what we find in BrCa, dN/dS and dMF/dLF are statistically significantly higher in globally expressed compared to non-globally expressed genes (*P*<0.05) in 8, 13, and 9 of the 13 additional tumor types, for dN/dS, dMF/dLF(SIFT), and dMF/dLF(polyphen) respectively. Strikingly, even when this difference is not strong enough to be statistically significant, dN/dS and dMF/dLF are always higher for globally compared to non-globally expressed genes, except for two cases in which they are equal (Table 2). Thus, our finding of higher somatic functional variation within globally expressed genes extends to many, if not all cancer types.

**Table 2:** Higher dN/dS and dMF/dLF values for globally, compared to non-globally expressed genes in 13 additional cancer types.

		Non-synonymous vs. synonymous				SIFT				Polyphen-2			
Cancer^a^	Globally expressed	# non-syn^b^	# syn^c^	dN/dS	Significant difference	# MF	# LF	dMF/dLF	Significant difference	# MF	# LF	dMF/dLF	Significant difference
BLCA	Yes	10008	3738	0.79	Yes	5721	4179	1.91	Yes	5169	4504	0.98	Yes
	No	15105	6507	0.70		8087	6606	1.63		7248	7066	0.92	
COAD	Yes	22212	10896	0.60	No	12531	9550	1.83	Yes	11251	10191	0.94	Yes
	No	43179	22109	0.59		22065	20515	1.44		19599	20981	0.83	
GBM	Yes	3926	1404	0.82	Yes	2306	1580	2.03	Yes	2106	1650	1.09	Yes
	No	10091	4041	0.75		5293	4398	1.61		4584	4626	0.88	
HNSC	Yes	12871	4661	0.81	Yes	7541	5196	2.02	Yes	6743	5537	1.04	Yes
	No	24719	10007	0.74		13664	10273	1.77		12036	10880	0.99	
KIRC	Yes	8600	3119	0.81	No	4859	3549	1.91	Yes	4453	3847	0.99	Yes
	No	13368	5090	0.79		7154	5767	1.66		6214	6180	0.90	
LUAD	Yes	31215	10392	0.89	Yes	13664	9007	2.11	Yes	16701	13267	1.07	No
	No	80079	28938	0.83		33323	23390	1.90		40593	33862	1.07	
LUSC	Yes	13014	4647	0.83	No	7637	5262	2.02	Yes	6897	5620	1.05	No
	No	28855	10788	0.81		16462	11901	1.85		14539	12530	1.04	
OV	Yes	6196	1884	0.97	Yes	694	1212	0.80	Yes	1533	1250	1.04	No
	No	10954	3697	0.89		1128	2255	0.67		2612	2304	1.01	
READ	Yes	6873	2229	0.91	Yes	3999	2835	1.97	Yes	3664	2970	1.05	Yes
	No	12988	4953	0.79		7076	5779	1.63		6227	5976	0.93	
SKCM	Yes	30030	16236	0.55	No	18112	11630	2.17	Yes	14825	12085	1.04	Yes
	No	91840	50015	0.55		50791	38809	1.75		42835	40112	0.95	
STAD	Yes	24671	10017	0.73	No	14321	10164	1.96	Yes	12984	10945	1.01	No
	No	44461	18847	0.71		24621	18808	1.75		22150	19741	1.00	
THCA	Yes	2207	754	0.86	Yes	1364	821	2.31	Yes	1185	927	1.09	Yes
	No	3292	1352	0.73		1657	1426	1.55		1415	1592	0.79	
UCEC	Yes	39676	13266	0.88	Yes	23313	15909	2.04	Yes	20915	16232	1.10	Yes
	No	67448	24386	0.83		37068	28292	1.75		31898	28458	1.00	

a BLCA - Bladder Urothelial Carcinoma, COAD - Colon adenocarcinoma, GBM - Glioblastoma multiforme, HNSC - Head and Neck squamous cell carcinoma, KIRC - Kidney renal clear cell carcinoma, LUAD - Lung adenocarcinoma, LUSC - Lung squamous cell carcinoma, OV - Ovarian serous cystadenocarcinoma, READ - Rectum adenocarcinoma, SKCM - Skin Cutaneous Melanoma, STAD - Stomach adenocarcinoma, THCA - Thyroid carcinoma, UCEC - Uterine Corpus Endometrioid Carcinoma.

b Non – Synonymous.

c Synonymous.

### Known cancer genes and genes presenting a clear signal of positive selection are enriched for global expression patterns

**Figure 2:**
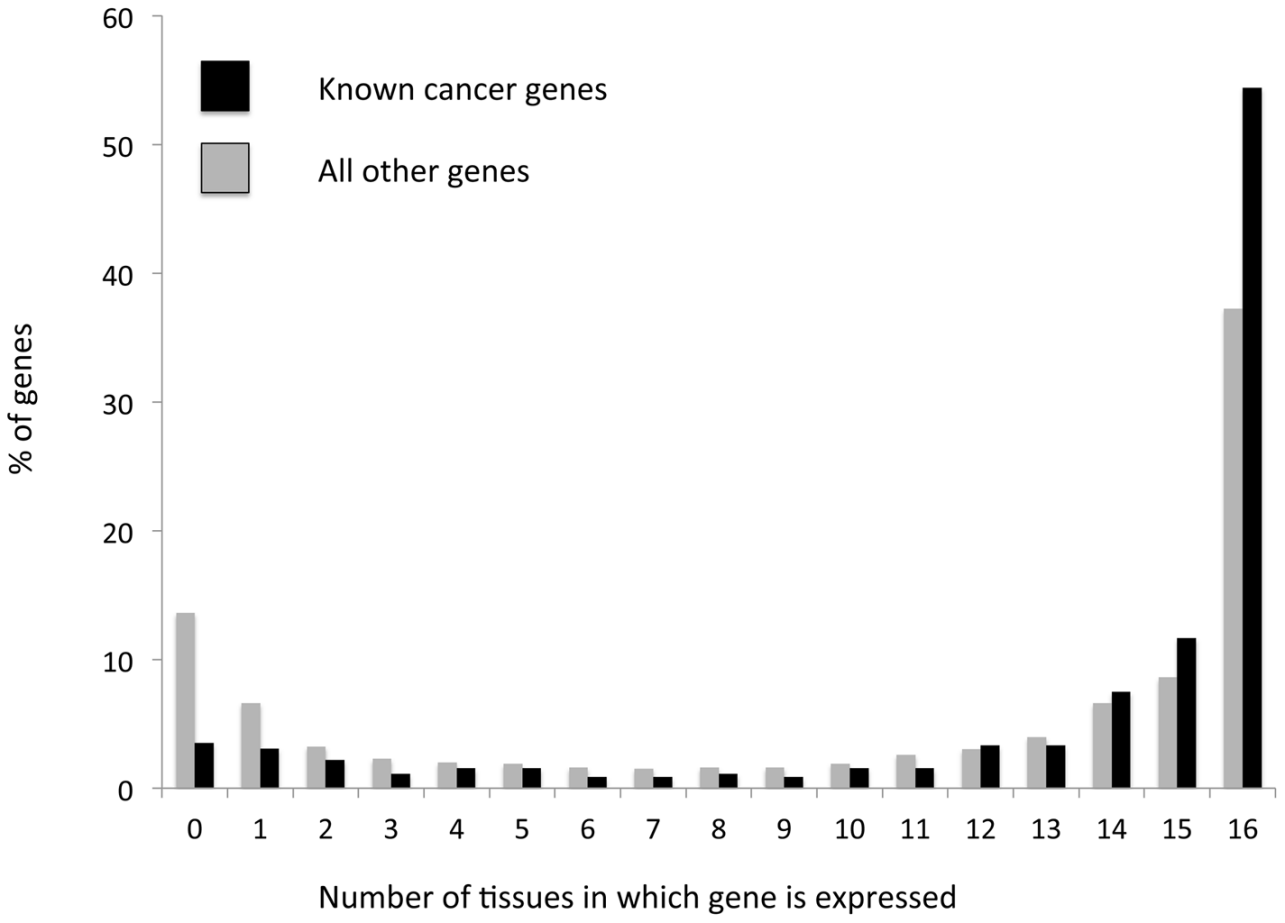
Cancer-associated genes tend to more frequently be globally expressed, and less frequently be expressed in a tissue specific manner than other genes. Genes that are known to be associated with cancer (black) and all remaining genes (gray) were grouped based on the number of tissues in which their expression has been detected (out of 16 examined tissues). The frequency of genes within each bin is depicted. Cancer genes display a significant (*P*<0.0001, according to a χ^2^ test) enrichment for global expression patterns (defined as expression across all 16 examined tissues). At the same time, cancer associated genes are ∼2.5 times less likely than other genes to not be expressed in any tissue, or be expressed in a tissue specific manner (1–3 tissues, a significant depletion, *P*<0.0001).

To further support our conclusion that increased functional variation within globally expressed genes stems from increased positive selection acting on these genes within tumors, we examined the expression patterns of a group of known cancer genes, contained in the ‘cancer gene census’ database [33]. As discussed above these known cancer genes are expected to be subject to positive selection in cancer evolution, as they carry mutations that allow cells to gain functions important for cancer. We found that known cancer genes tend to be significantly more globally expressed than other genes (*P*<<0.0001 according to a χ2 test, Figure 2). More than half (∼54%) of known cancer-associated genes are expressed across all 16 examined tissues (∼1.5 fold higher than for genes with no known cancer function); a minority (∼10%) of known cancer associated genes are expressed in three or less tissues (∼2.5-fold lower than for non-cancer associated genes, a significant depletion (*P*<<0.0001), Figure 2). This result provides further support for positive selection affecting globally expressed genes more strongly within tumors, as it demonstrates that genes that are known to be under positive selection within tumors tend more often to be globally expressed.

We also examined the expression patterns of genes for which we could observe a clear signal of positive selection (dN/dS significantly higher than 1, *P*<0.05 see above). We identified 104 such genes, when combining data of cancer somatic substitutions from all tumors types covered by the TCGA, for which no publication restrictions apply (Table S2). For 98 of these genes there was expression data available, and of these genes 50 (51%) are globally expressed across all 16 tissues examined (Table S3). This is a weakly significant enrichment (*P* = 0.03) compared to what is observed for genes that do not have dN/dS values significantly higher than 1 (40.2% globally expressed). The enrichment in global expression patterns becomes stronger when a higher significance is required for dN/dS being higher than 1. Of the 15 genes for which dN/dS is higher than 1 with a significance lower than 0.001, 12 are globally expressed (80%, significant enrichment compared to genes with dN/dS not significantly higher than 1, *P* = 0.004). Furthermore, when considering the four genes we identified as having dN/dS higher than 1 in the BrCa somatic dataset, all four of these genes are globally expressed (Table S4). Thus, genes with a clear signal of positive selection of dN/dS significantly higher than 1 are significantly enriched for global expression patterns. This again supports our conclusion that positive selection more often affects globally expressed genes.

### Globally expressed genes not yet associated with cancer are also enriched for functional BrCa somatic substitutions

To investigate whether the signal of stronger positive selection on globally expressed genes stems only from the fact that known cancer genes are more often globally expressed, we repeated our analyses after removing all known cancer genes from consideration. Interestingly, even when we remove known cancer genes from our analyses, we find that dN/dS and dMF/dLF are significantly higher on globally compared to non-globally expressed genes (Table 1, *P* ranges between 0.015 and <<0.0001 for all three dN/dS and dMF/dLF comparisons). This suggests that significant positive selection acts on globally expressed genes that have not yet been implicated in cancer, suggesting that there are a substantial number of yet undiscovered globally expressed genes carrying mutations that can confer a growth advantage on tumor cells.

## Discussion

Our results demonstrate that the proportion of functional variation is much higher within somatic cancer substitutions compared to germline substitutions. These results indicate that natural selection affects somatic mutations within tumors in a different manner than it affects germline mutations. Specifically, patterns of somatic substitutions within tumors are affected much less by purifying selection, compared to patterns of germline variation between different humans. The strong effects of purifying selection on patterns of germline substitution make it difficult to observe positive selection when examining patterns of variation within and between species. Observing positive selection on somatic substitutions within tumors is much easier, likely both because purifying selection affects a much smaller proportion of mutations, and because positive selection affects a much higher proportion of mutations.

As discussed above, purifying selection on cancer somatic mutations is likely relaxed due to a combination of factors, including the effects of hitchhiking [32], the fact that somatic mutations affect only a small subset of cells while germline substitutions affect the entire organism, and small effective population sizes of stem cell pools. At the same time, it seems reasonable that positive selection would affect a larger proportion of somatic cancer mutations compared to germline mutations. Organisms tend to be well adapted and thus close to their optimum fitness. Most mutations that affect fitness will reduce rather than increase the fitness of a well-adapted organism [9]. In contrast cancer cells may be far from their fitness optimum when it comes to their ability to replicate independently, avoid organismal defenses, maintain themselves, protect themselves against chemotherapies and eventually invade other tissues. Therefore, it is likely that a much higher proportion of functional mutations would have an advantageous effect on a cancer cell compared to an organism. Furthermore, the microenvironment of cancer cells is thought to be highly dynamic [37], [38], both due to the effects of the cancer on its environment (e.g. acidification [39]), and due to host attempts to combat the cancer [40]. Populations of microbes exposed to novel environments have been shown to experience increased positive selection [41], and it is reasonable that tumor cells would experience a similar effect.

In our analyses we assumed that dMF/dLF would increase under positive selection. Such an assumption may be violated if mutations within the LF category of sites are more likely to be moderately functional than mutations at the MF category of sites, and if fitness is near an optimum. Under a scenario of nearly optimal fitness, adaptation may advance via small steps (i.e. through mutations with moderate effects). If such mutations are enriched within the LF category of sites positive selection may then increase dLF relative to dMF and reduce dMF/dLF. In light of this possibility and in order to examine whether it was reasonable for us to expect dMF/dLF to increase under positive selection within tumors we compared dMF/dLF of BrCa somatic substitutions within known cancer genes (which we know are under positive selection) to dMF/dLF within all remaining genes. We find that fitting with positive selection increasing dMF/dLF within tumors, known cancer genes have significantly higher values of dMF/dLF (SIFT) and dMF/dLF (Polyphen) than the remainder of genes (P<<0.0001 for both comparisons, Table 1). These results support our assumption that dMF/dLF increases under positive selection within tumors, and also supports the idea that adaptation within tumors occurs far from a fitness optimum.

Globally expressed genes are enriched for important housekeeping functions and more likely to be predicted as essential, compared to genes that are not globally expressed. In the germline, globally expressed genes are significantly more strongly affected by purifying selection than non-globally expressed genes, leading to lower levels of non-synonymous variation within globally expressed genes. In sharp contrast to this, functional somatic variation is increased in globally expressed genes, compared to non-globally expressed genes within tumors. This is very unlikely to be the result of less constraint acting on globally expressed genes within tumors. After all, there is no reason to believe that genes enriched for housekeeping and essential functions will suddenly be significantly less important within tumors than other genes. Therefore, higher levels of cancer somatic functional variation within globally expressed genes compared to genes not expressed in breast very likely reflects increased positive selection on globally expressed genes, rather than relaxed purifying selection. Supporting this conclusion we can further demonstrate that known cancer genes as well as genes showing a clear signal of positive selection of dN/dS of cancer somatic substitutions significantly greater than 1 are enriched for global expression patterns.

We compared levels of BrCa functional variation between globally expressed genes and two groups of non-globally expressed genes: those that are not expressed in breast and those that are. We found a higher enrichment in BrCa somatic functional variation when globally expressed genes were compared to genes that are not expressed in breast. It is extremely unlikely that globally expressed genes would be evolving under less constraint within breast, than genes that are not at all expressed in breast. This provides further support for our conclusion that increased functional variation in globally expressed genes stems from increased positive selection rather than from relaxed purifying selection. At the same time, we also observed a significant enrichment in BrCa somatic functional variation of globally expressed genes, compared to genes that are not globally expressed but are nevertheless expressed in breast. This indicates the increased positive selection on globally expressed genes within breast tumors does not stem solely from the fact that such globally expressed genes are expressed in breast. Rather, it is likely that at least part of the reason for the increased positive selection on globally expressed genes is their enrichment for important housekeeping and essential functions. Our results therefore indicate that changes required for cancer development, maintenance and progression tend to occur in genes that carry out the most basic and important cellular functions. These results fit well with previous results that have demonstrated that cancer genes tend to evolve under more constraint in the germline, compared to other genes [42].

Even when we remove from consideration those genes that we already know are involved in cancer (and which tend to more frequently have global expression patterns), we still detect a strong enrichment in BrCa somatic functional variation within globally expressed, compared to non-globally expressed genes. This strongly suggests that globally expressed genes are enriched for yet undiscovered cancer related functions, and that it would be wise to pay special attention to globally expressed genes when searching for novel cancer genes.

The approach we used to demonstrate that globally expressed genes are evolving under more pervasive positive selection than non-globally expressed genes can be applied to other groups of genes. Our approach works by classifying groups of genes based on their predicted importance, and then finding instances in which genes that are expected to be more important and therefore evolving under more constraint are nevertheless enriched for functional substitutions. We expect that very soon data of somatic substitutions within tumors will be abundant enough to allow us to modify our approach to identify individual genes that are evolving under positive selection within tumors. Identifying such cancer related genes is a major goal of cancer genomics.

Many of the positively selected mutations within tumors may confer a relatively small fitness advantage during cancer development, acting as “mini-drivers” of cancer. Classical methods that rely on a strong phenotypic effect of cancer mutations (e.g. gene transfer) may not be able to identify such mini-drivers. Yet, such moderately advantageous mutations may still play an important role in cancer development and progression. The approach described here could provide a framework for detecting mini-drivers and for detecting drivers with larger fitness effects, both of which will be essential for understanding the evolution of cancers and designing therapies to exploit their weaknesses.

## Materials and Methods

### Data sources

Data of breast cancer somatic substitutions from tumors of 772 patients were extracted from the Cancer Genome Atlas project (TCGA) [31] on March 8^th^ 2013. Data of somatic substitutions from all additional 15 cancer types for which no publication restrictions apply as of the end of December 2013 (Table S2) were downloaded from the TCGA on July 22^nd^ 2013. The data was then parsed to count only once any duplicated substitution that appears more than once within a single tumor. Somatic substitutions were identified by TCGA through sequencing of tumors and healthy tissues of the same individuals. Since variable sites appearing within each tumor would have to be present at relatively high frequency within the tumor in order to be detected, such variable sites had time to be affected by natural selection. It is therefore possible to characterize the intensity with which purifying and positive selection act on somatic mutations within tumors by examining dN/dS and dMF/dLF of the substitutions found in these tumors.

Data of germline substitutions were extracted from the 1000 human genome project [11] (Phase 1, V3, latest version as of May 2013). To determine whether each of these germline substitutions were coding or not, and if coding whether they were non-synonymous or synonymous, we used the SnpEff program [43]. This resulted in 512,903 coding non-synonymous or synonymous substitutions, 36,167 of them appearing at a frequency of higher than 0.1.

Gene expression data were extracted as described in TissueNet [44]. Data of gene expression across tissues were extracted from Su *et al*.[45] and Illumina Body Map 2.0 [46]. Genes with intensity value above 100 [47] or at least 1RPKM were considered as expressed. Matching tissues were consolidated manually.

In order to parse the different datasets, gene name conversion tables were extracted from ENSEMBL [48], the HUGO Gene Nomenclature Committee (HGNC) [49], and the Genecards database [50].

A list of the genes currently known to be associated with cancer was downloaded from the Catalogue of Somatic Mutations in Cancer (COSMIC) [33]

Data of predicted gene essentiality was extracted from [35]. In this study essentiality of human genes was predicted according to whether their orthologs in mice were essential.

### Calculating dN/dS

Somatic substitutions or germline substitutions within protein coding genes were classified as non-synonymous or synonymous. For each gene we considered only the longest possible transcript (so as not to count single substitutions within a single patient twice). It is expected that following the inactivation of a gene through a nonsense or frameshift mutation, subsequent mutations within that gene, within that same tumor, may not be under selection. For this reason, we removed from consideration somatic substitutions from a certain gene in a certain patient if a nonsense or frameshift substitution was found in the same gene in the same patient. This left us with 41,657 non-synonymous or synonymous coding somatic substitutions. (Note that results reported in this paper remained entirely stable even when we did not perform this clean up step).

Unlike in germline evolution, somatic substitutions at the same site, and with the same nucleotide change, have to occur repeatedly in order to be seen in more than one individual. Therefore we counted substitutions as many times as they appeared within the TCGA data.

A script was written to calculate the number of synonymous and non-synonymous sites within each human protein-coding gene. These calculations were carried out as in [15]. Briefly, we calculated for each protein-coding DNA site the proportion of changes that would be non-synonymous (alter the amino-acid sequence of the encoded protein), and the proportion of changes that would be synonymous. We then added up these proportions across the gene to obtain the proportion of non-synonymous and synonymous sites. The sum of these two proportions is the length of the considered gene. Data regarding the numbers of non-synonymous and synonymous sites are summarized in Table S6.

Once we know the number of non-synonymous substitutions (n), and synonymous substitutions (s) that have occurred within a group of genes in a group of breast tumors, and we also know how many non-synonymous sites (N), and synonymous sites (S) there are within these genes, we can calculate the ratio of the rates of non-synonymous and synonymous substitutions (dN/dS) for that group of genes, in the considered tumors, as:
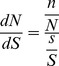



The exact same approach was used to calculate dN/dS for germline substitutions.

### Calculating dMF/dLF

#### SIFT

The SIFT algorithm classifies non-synonymous substitutions into those more or less likely to be functional (or as they refer to it ‘damaging’), based on considerations of conservation [21]. In order to calculate the number of more functional and less functional sites, based on SIFT predictions, we downloaded the SIFT predictions for all human proteins from the SIFT webserver (http://sift.jcvi.org/). For each protein-coding gene we considered only the longest possible transcript (to avoid counting potential substitutions more than once). For each protein position, the SIFT predictions give a “potential functionality” score for each of the 19 possible amino acid alterations at that position. Following the recommendation of the algorithm's authors [21] we consider any change with a score of ≤0.05 to be ‘damaging’, or as we consider it more likely to be functional (MF). Changes with a score of >0.05 are considered less likely to be functional (LF). Using the data provided by SIFT we assigned each possible change as either more or less functional. Possible changes are defined as those changes that could occur via single base mutation. Based on this we could calculate for each codon the proportion of possible changes that would be more and less functional. We then added up these proportions across the gene to obtain the proportion of more function (MF) and less functional (LF) sites, within that gene. Data regarding the numbers of SIFT MF and LF sites are summarized in Table S6.

SIFT predictions were also obtained for all of the non-synonymous substitutions contained in the TCGA breast dataset, and for the germline substitutions extracted from the 1000 human genome project. This allows us to calculate dMF/dLF(SIFT) as:
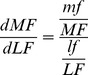



Where mf and lf are the number of more likely to be functional and less likely to be functional substitutions, respectively, within that group of genes, and MF and LF, are the number of more likely to be functional, and less likely to be functional sites, respectively within that group of genes.

#### Polyphen-2

Polyphen-2 classifies positions into more or less likely to be functional based on sequence and structure considerations [22]. Predictions of potential functionality were downloaded from the Polyphen-2 webserver (http://genetics.bwh.harvard.edu/pph2/dokuwiki/downloads). These predictions give for each possible nucleotide substitution in each coding nucleotide site a classification. Possible substitutions are defined as those amino acid substitutions that can be achieved via a single nucleotide change. Possible substitutions, and actual cancer somatic substitutions were considered to be MF if they were classified as ‘possibly damaging’ or ‘probably damaging’ by Polyphen-2. Possible substitutions or actual cancer somatic substitutions were considered to be LF if they were classified by Polyphen-2 to be ‘benign’. Based on these classifications, the numbers of MF and LF sites were calculated per gene, by summing up the numbers of MF and LF predictions across that gene, and dMF/dLF was then calculated as described above for SIFT. Data regarding the numbers of Polyphen MF and LF sites are summarized in Table S6.

### Significance calculations

#### Calculating whether dN/dS and dMF/dLF values differed significantly from one

The distribution of substitutions from each category was compared to the distribution of sites within the same category using a χ^2^ test.

#### Testing for significance of differences in dN/dS (or dMF/dLF) between globally and non-globally expressed genes

a χ^2^ test was used to compare the numbers of non-synonymous and synonymous substitutions in globally expressed genes (n_global,_ s_global_) to the numbers of non-synonymous and synonymous substitutions in non-globally expressed genes (n_non-global_, s_non-global_). Before carrying out this comparison, we had to account for differences in the distribution of substitutions that were due to possible differences in the distribution of non-synonymous and synonymous sites between the two gene groups. For example, it is possible that one would see a higher proportion of non-synonymous substitutions in globally expressed genes, simply because there is a higher proportion of non-synonymous sites within these genes. To correct for this possibility, we divided n_global_ by a correction factor (cf) that corrects for differences in the distribution of non-synonymous (N), and synonymous (S) sites between the globally and non-globally expressed genes.
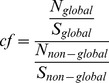



Similar corrections and significance calculations were carried out when examining whether dMF/dLF of globally expressed genes were significantly higher than for non-globally expressed genes, and when comparing other groups of genes.

## Supporting Information

Table S1 Much higher proportion of functional variation in BrCa somatic substitutions compared to germline substitutions.
(DOCX)

Table S2 Summary of cancer types analyzed.
(DOCX)

Table S3 List of genes for which dN/dS of cancer somatic substitutions (combined across 16 cancer types) is significantly higher than 1.
(DOCX)

Table S4 List of genes for which dN/dS of breast cancer somatic substitutions is significantly higher than 1.
(DOCX)

Table S5 Genes expressed in 14–16 tissues are enriched for functional BrCa somatic substitutions compared to genes that are expressed in less than 14 tissues.
(DOCX)

Table S6 Summary of numbers of sites.
(DOCX)
