# Supplementary material for: Cancer Evolution Is Associated with Pervasive Positive Selection on Globally Expressed Genes
Source: PLoS Genet. 2014 Mar 6;10(3):e1004239. doi: 10.1371/journal.pgen.1004239 (PMC3945297; doi:10.1371/journal.pgen.1004239)
Supplement: Table S1 — Much higher proportion of functional variation in BrCa somatic substitutions compared to germline substitutions. (DOCX) [file pgen.1004239.s001.docx]

**Table S1.** Much higher proportion of functional variation in BrCa somatic substitutions compared to germline substitutions

|  | Non-synonymous vs. synonymous | | | SIFT | | | Polyphen | | |
| --- | --- | --- | --- | --- | --- | --- | --- | --- | --- |
|  | # non-syn | # syn | dN/dS | # MF | # LF | dMF/dLF | # MF | # LF | dMF/dLF |
| Germline | 302469 | 210434 | 0.43 | 130732 | 171089 | 1.03 | 103234 | 172947 | 0.52 |
| Germline (frequency > 0.1)^a^ | 17325 | 18812 | 0.28 | 3499 | 13553 | 0.35 | 2211 | 12441 | 0.16 |
| Somatic BrCa | 30535 | 11122 | 0.82 | 15854 | 12886 | 1.66 | 13569 | 12656 | 0.94 |

^a^We consider separately germline substitutions occurring at a higher frequency of >0.1 in the human population, because germline substitutions appearing at lower frequencies are expected to be less affected by natural selection than germline substitutions appearing at high frequencies [[50](#_ENREF_50)]. This is because rare polymorphisms have not yet had time to be strongly affected by selection and therefore still contain many deleterious substitutions that with time would be removed from the population.
