# Supplementary material for: Cancer Evolution Is Associated with Pervasive Positive Selection on Globally Expressed Genes
Source: PLoS Genet. 2014 Mar 6;10(3):e1004239. doi: 10.1371/journal.pgen.1004239 (PMC3945297; doi:10.1371/journal.pgen.1004239)
Supplement: Table S2 — Summary of cancer types analyzed. (DOCX) [file pgen.1004239.s002.docx]

**Table S2**. Summary of cancer types analyzed

| Cancer type | Abbreviation | # of sequenced samples | # of protein-coding non-synonymous and synonymous somatic substitutions |
| --- | --- | --- | --- |
| Bladder Urothelial Carcinoma | BLCA | 136 | 36821 |
| Breast invasive carcinoma | BRCA | 772 | 41648 |
| Colon adenocarcinoma | COAD | 268 | 102391 |
| Glioblastoma multiforme | GBM | 291 | 20706 |
| Head and Neck squamous cell carcinoma | HNSC | 323 | 54795 |
| Kidney Chromophobe | KICH | 65 | 1531 |
| Kidney renal clear cell carcinoma | KIRC | 65 | 1531 |
| Acute Myeloid Leukemia | LAML | 72 | 794 |
| Lung adenocarcinoma | LUAD | 452 | 31657 |
| Lung squamous cell carcinoma | LUSC | 72 | 794 |
| Ovarian serous cystadenocarcinoma | OV | 518 | 158775 |
| Rectum adenocarcinoma | READ | 177 | 59986 |
| Skin Cutaneous Melanoma | SKCM | 269 | 197269 |
| Stomach adenocarcinoma | STAD | 230 | 102104 |
| Thyroid carcinoma | THCA | 424 | 8133 |
| Uterine Corpus Endometrioid Carcinoma | UCEC | 248 | 152198 |
