# Supplementary material for: Cancer Evolution Is Associated with Pervasive Positive Selection on Globally Expressed Genes
Source: PLoS Genet. 2014 Mar 6;10(3):e1004239. doi: 10.1371/journal.pgen.1004239 (PMC3945297; doi:10.1371/journal.pgen.1004239)
Supplement: Table S3 — List of genes for which dN/dS of cancer somatic substitutions (combined across 16 cancer types) is significantly higher than 1. (DOCX) [file pgen.1004239.s003.docx]

Table S3. List of genes for which dN/dS of cancer somatic substitutions (combined across 16 cancer types) is significantly higher than 1

| Gene Name | Ensembl ID | Number of Tissues in which gene is expressed | Cancer-associated^a^ | dN/dS | P-Value^b^ |
| --- | --- | --- | --- | --- | --- |
| TP53 | ENSG00000141510 | 16 | Y | 15.04 | 1.90E-50 |
| PIK3CA | ENSG00000121879 | 16 | Y | 10.79 | 9.07E-36 |
| BRAF | ENSG00000157764 | 16 | Y | 8.00 | 8.41E-24 |
| KRAS | ENSG00000133703 | 16 | Y | 11.39 | 4.15E-15 |
| IDH1 | ENSG00000138413 | 16 | Y | 8.79 | 4.66E-11 |
| PTEN | ENSG00000171862 | 16 | Y | 6.58 | 4.69E-08 |
| NRAS | ENSG00000213281 | 16 | Y | 8.64 | 7.65E-08 |
| FAM194B | ENSG00000165837 | 1 | N | 10.59 | 6.07E-07 |
| NFE2L2 | ENSG00000116044 | 16 | Y | 9.39 | 3.77E-06 |
| CTNNB1 | ENSG00000168036 | 16 | Y | 2.76 | 2.00E-05 |
| FBXW7 | ENSG00000109670 | 16 | Y | 3.10 | 1.05E-04 |
| VHL | ENSG00000134086 | 16 | Y | 3.45 | 1.55E-04 |
| SMAD4 | ENSG00000141646 | 16 | Y | 3.65 | 4.96E-04 |
| CDKN2A | ENSG00000147889 | 7 | Y | 3.08 | 8.19E-04 |
| MUC7 | ENSG00000171195 | 4 | N | 2.52 | 8.34E-04 |
| KLHL4 | ENSG00000102271 | 11 | N | 2.41 | 1.10E-03 |
| TRA2B | ENSG00000136527 | 16 | N | 13.00 | 1.13E-03 |
| FRG1B | ENSG00000149531 | 16 | N | 1.84 | 1.54E-03 |
| PTPLA | ENSG00000165996 | 0 | N | 5.29 | 1.98E-03 |
| RPSAP58 | ENSG00000205246 | 6 | N | 3.49 | 2.23E-03 |
| LOC100288822 | ENSG00000182327 | 2 | N | 10.77 | 3.70E-03 |
| SPOP | ENSG00000121067 | 16 | N | 4.61 | 5.14E-03 |
| TSGA14 | ENSG00000106477 | 7 | N | 5.97 | 5.36E-03 |
| RGPD5 | ENSG00000015568 | 0 | N | 3.38 | 5.58E-03 |
| SETD2 | ENSG00000181555 | 16 | Y | 1.84 | 8.49E-03 |
| ERBB3 | ENSG00000065361 | 8 | N | 1.88 | 8.67E-03 |
| CENPC1 | ENSG00000145241 | 15 | N | 4.11 | 1.04E-02 |
| TAF1 | ENSG00000147133 | 16 | N | 1.81 | 1.12E-02 |
| COL5A2 | ENSG00000204262 | 14 | N | 1.58 | 1.30E-02 |
| KEAP1 | ENSG00000079999 | 16 | N | 1.79 | 1.32E-02 |
| XRN1 | ENSG00000114127 | 16 | N | 2.05 | 1.36E-02 |
| FAM184A | ENSG00000111879 | 12 | N | 2.33 | 1.36E-02 |
| PRB2 | ENSG00000121335 | 0 | N | 1.83 | 1.38E-02 |
| KIAA0907 | ENSG00000132680 | 16 | N | 2.47 | 1.39E-02 |
| RTN4 | ENSG00000115310 | 16 | N | 2.76 | 1.41E-02 |
| Unknown | ENSG00000183793 | 0 | N | 3.38 | 1.42E-02 |
| ABI3BP | ENSG00000154175 | 0 | N | 1.89 | 1.46E-02 |
| COL1A2 | ENSG00000164692 | 15 | N | 1.48 | 1.51E-02 |
| HNRNPH2 | ENSG00000268642 | No data | N | 4.93 | 1.55E-02 |
| PARP2 | ENSG00000129484 | 16 | N | 4.92 | 1.55E-02 |
| EPS8 | ENSG00000151491 | 16 | N | 3.30 | 1.56E-02 |
| CSNK2A1 | ENSG00000101266 | 16 | N | 3.32 | 1.57E-02 |
| TFEC | ENSG00000105967 | 4 | N | 3.87 | 1.59E-02 |
| MAX | ENSG00000125952 | 16 | N | 4.97 | 1.60E-02 |
| ZNF492 | ENSG00000229676 | 0 | N | 2.70 | 1.66E-02 |
| AADACL2 | ENSG00000197953 | 0 | N | 2.92 | 1.78E-02 |
| PRB4 | ENSG00000230657 | 0 | N | 2.57 | 1.78E-02 |
| EVI2B | ENSG00000185862 | 16 | N | 4.80 | 1.80E-02 |
| HRAS | ENSG00000174775 | 16 | Y | 2.94 | 1.88E-02 |
| CMAS | ENSG00000111726 | 16 | N | 7.63 | 1.90E-02 |
| PIK3C3 | ENSG00000078142 | 16 | N | 2.48 | 1.92E-02 |
| OR8U8 | ENSG00000262315 | No data | N | 3.19 | 2.05E-02 |
| CTAGE9 | ENSG00000236761 | 12 | N | 7.45 | 2.06E-02 |
| SOS1 | ENSG00000115904 | 16 | N | 1.97 | 2.07E-02 |
| MTX1 | ENSG00000173171 | 16 | N | 4.67 | 2.12E-02 |
| REST | ENSG00000084093 | 15 | N | 2.22 | 2.13E-02 |
| GORASP2 | ENSG00000115806 | 16 | N | 2.82 | 2.34E-02 |
| HRCT1 | ENSG00000196196 | 11 | N | 7.40 | 2.36E-02 |
| ZNF844 | ENSG00000223547 | 0 | N | 1.94 | 2.43E-02 |
| PHIP | ENSG00000146247 | 15 | N | 1.79 | 2.44E-02 |
| TSPYL1 | ENSG00000189241 | 16 | N | 7.17 | 2.45E-02 |
| KDM4A | ENSG00000066135 | 16 | N | 2.55 | 2.47E-02 |
| PRKRIR | ENSG00000137492 | 16 | N | 2.75 | 2.53E-02 |
| 7-Mar | ENSG00000136536 | 16 | N | 3.05 | 2.55E-02 |
| MTOR | ENSG00000198793 | 16 | N | 1.46 | 2.62E-02 |
| METTL14 | ENSG00000145388 | 16 | N | 4.41 | 2.67E-02 |
| WDR17 | ENSG00000150627 | 0 | N | 1.68 | 2.79E-02 |
| COL11A1 | ENSG00000060718 | 1 | N | 1.32 | 2.91E-02 |
| COL21A1 | ENSG00000124749 | 14 | N | 1.68 | 3.03E-02 |
| STXBP5L | ENSG00000145087 | 13 | N | 1.56 | 3.03E-02 |
| FGFR2 | ENSG00000066468 | 14 | Y | 1.67 | 3.09E-02 |
| LCE2D | ENSG00000187223 | No data | N | 6.86 | 3.11E-02 |
| FAM122C | ENSG00000156500 | 12 | N | 6.77 | 3.13E-02 |
| IFIT2 | ENSG00000119922 | 2 | N | 4.25 | 3.14E-02 |
| TMEM244 | ENSG00000203756 | 0 | N | 6.70 | 3.34E-02 |
| B2M | ENSG00000166710 | 16 | N | 6.71 | 3.34E-02 |
| IDH2 | ENSG00000182054 | 16 | Y | 4.20 | 3.36E-02 |
| GK2 | ENSG00000196475 | 1 | N | 1.78 | 3.41E-02 |
| ST7 | ENSG00000004866 | 16 | N | 1.98 | 3.43E-02 |
| GPSM2 | ENSG00000121957 | 16 | N | 2.89 | 3.45E-02 |
| ZNF345 | ENSG00000251247 | 14 | N | 2.60 | 3.56E-02 |
| ITGB1BP2 | ENSG00000147166 | 8 | N | 4.13 | 3.66E-02 |
| PRDX6 | ENSG00000117592 | 16 | N | 6.45 | 3.73E-02 |
| PTH2 | ENSG00000142538 | 0 | N | 4.20 | 3.93E-02 |
| REV3L | ENSG00000009413 | 16 | N | 1.61 | 3.95E-02 |
| CASP8 | ENSG00000064012 | 16 | N | 2.12 | 4.27E-02 |
| C8orf47 | ENSG00000177459 | 14 | N | 3.96 | 4.36E-02 |
| Unknown | ENSG00000263563 | No data | N | 1.83 | 4.37E-02 |
| BRWD3 | ENSG00000165288 | 16 | N | 1.51 | 4.43E-02 |
| ZMAT4 | ENSG00000165061 | 2 | N | 3.19 | 4.47E-02 |
| FBXO22 | ENSG00000167196 | 16 | N | 2.78 | 4.47E-02 |
| KIFC3 | ENSG00000269180 | No data | N | 2.76 | 4.62E-02 |
| NAP1L2 | ENSG00000186462 | 12 | N | 1.96 | 4.68E-02 |
| KRTAP4-5 | ENSG00000198271 | 0 | N | 2.53 | 4.68E-02 |
| HLA-DRA | ENSG00000230726 | 11 | N | 2.76 | 4.73E-02 |
| INTS6 | ENSG00000102786 | 16 | N | 2.48 | 4.73E-02 |
| PIK3CB | ENSG00000051382 | 16 | N | 1.84 | 4.77E-02 |
| UBN2 | ENSG00000157741 | 15 | N | 1.85 | 4.78E-02 |
| KIR2DL1 | ENSG00000262556 | No data | N | 1.96 | 4.84E-02 |
| ACAD11 | ENSG00000240303 | 0 | N | 2.72 | 4.86E-02 |
| GEN1 | ENSG00000178295 | 4 | N | 2.46 | 4.87E-02 |
| CCNE2 | ENSG00000175305 | 14 | N | 3.10 | 4.89E-02 |
| POTED | ENSG00000166351 | 0 | N | 3.09 | 4.92E-02 |
| CLDND1 | ENSG00000080822 | 16 | N | 5.90 | 5.00E-02 |

^a^According to the cancer Gene Census database

^b^P-Value with which the null hypothesis stating that dN/dS is not different from 1 can be rejected according to a χ^2^ test.
