# Supplementary material for: Cancer Evolution Is Associated with Pervasive Positive Selection on Globally Expressed Genes
Source: PLoS Genet. 2014 Mar 6;10(3):e1004239. doi: 10.1371/journal.pgen.1004239 (PMC3945297; doi:10.1371/journal.pgen.1004239)
Supplement: Table S4 — List of genes for which dN/dS of breast cancer somatic substitutions is significantly higher than 1. (DOCX) [file pgen.1004239.s004.docx]

Table S4. List of genes for which dN/dS of breast cancer somatic substitutions is significantly higher than 1

| Gene name | Ensembl ID | Number of Tissues in which gene is expressed | Cancer-associated^a^ | BrCa -associated^a^ | dN/dS | P-Value^b^ |
| --- | --- | --- | --- | --- | --- | --- |
| PIK3CA | ENSG00000121879 | 16 | Yes | Yes | 17.97 | 4.23E-15 |
| TP53 | ENSG00000141510 | 16 | Yes | Yes | 23.57 | 1.54E-10 |
| TTN | ENSG00000155657 | 16 | No | No | 1.65 | 2.21E-02 |
| MLL3 | ENSG00000055609 | 16 | Yes | No | 3.92 | 4.42E-02 |

^a^According to the cancer Gene Census database

^b^P-Value with which the null hypothesis stating that dN/dS is not different from 1 can be rejected according to a χ^2^ test.
