# Supplementary material for: Cancer Evolution Is Associated with Pervasive Positive Selection on Globally Expressed Genes
Source: PLoS Genet. 2014 Mar 6;10(3):e1004239. doi: 10.1371/journal.pgen.1004239 (PMC3945297; doi:10.1371/journal.pgen.1004239)
Supplement: Table S5 — Genes expressed in 14–16 tissues are enriched for functional BrCa somatic substitutions compared to genes that are expressed in less than 14 tissues. (DOCX) [file pgen.1004239.s005.docx]

Table S5. Genes expressed in 14-16 tissues are enriched for functional BrCa somatic substitutions compared to genes that are expressed in less than 14 tissues

|  | Non-synonymous vs. synonymous | | | SIFT | | | Polyphen | | |
| --- | --- | --- | --- | --- | --- | --- | --- | --- | --- |
|  | # non-syn | # syn | dN/dS | # MF | # LF | dMF/dLF | # MF | # LF | dMF/dLF |
| Genes expressed in 14-16 tissues | 15931 | 5325 | 0.88 | 8794 | 6673 | 1.82 | 7768 | 6673 | 1.01 |
| Genes expressed in less than 14 tissues | 12736 | 5049 | 0.76 | 6376 | 5588 | 1.51 | 5406 | 5521 | 0.87 |
