# Supplementary material for: Cancer Evolution Is Associated with Pervasive Positive Selection on Globally Expressed Genes
Source: PLoS Genet. 2014 Mar 6;10(3):e1004239. doi: 10.1371/journal.pgen.1004239 (PMC3945297; doi:10.1371/journal.pgen.1004239)
Supplement: Table S6 — Summary of numbers of sites. (DOCX) [file pgen.1004239.s006.docx]

Table S6. Summary of numbers of sites^a^

|  | Non-synonymous vs. synonymous | | SIFT | | Polyphen | |
| --- | --- | --- | --- | --- | --- | --- |
|  | Non-syn | syn | MF | LF | MF | LF |
| All human genes | 29165351 | 8725204 | 3197645 | 4325205 | 22973551 | 20154713 |
| Genes expressed across all 16 examined tissues | 10101018 | 2978700 | 1227702 | 1710383 | 9097184 | 7746836 |
| Genes expressed in 0-15 of 16 examined tissues | 16394442 | 4934319 | 1917522 | 2558410 | 13627659 | 12159842 |
| Genes expressed in 0-15 of 16 examined tissues, including breast | 7811011 | 2329931 | 985775 | 1316575 | 7011714 | 6141378 |
| Genes expressed in 0-15 of 16 examined tissues, not including breast | 8583431 | 2604388 | 931747 | 1241835 | 6615945 | 6018464 |
| Genes expressed in 14-16 of 16 examined tissues | 14846900 | 4391296 | 1813717 | 2502997 | 13261888 | 11495869 |
| Genes expressed in 0-13 of 16 examined tissues | 11648560 | 3521723 | 1331507 | 1765796 | 9462955 | 8410809 |
| Genes expressed in 16 tissues with known cancer genes removed | 9603735 | 2833845 | 1168102 | 1627772 | 8549410 | 7310421 |
| Genes expressed in 0-15 tissues with known cancer genes removed | 16042703 | 4830616 | 1875365 | 2499725 | 13259387 | 11822922 |
| Cancer associated genes | 892389 | 261720 | 101831 | 141515 | 916046 | 773335 |
| Non cancer associated genes | 28272962 | 8463484 | 3095814 | 4183690 | 22057505 | 19381378 |

^a^Numbers of sites are rounded up or down to the closest integer
